# YOLOv8-CBAM: a study of sheep head identification in Ujumqin sheep

**DOI:** 10.3389/fvets.2025.1514212

**Published:** 2025-02-06

**Authors:** Qing Qin, Xingyu Zhou, Jiale Gao, Zhixin Wang, A. Naer, Long Hai, Suhe Alatan, Haijun Zhang, Zhihong Liu

**Affiliations:** ^1^Animal Science Department, Inner Mongolia Agricultural University, Hohhot City, China; ^2^Key Laboratory of Animal Genetics, Breeding and Reproduction in Inner Mongolia Autonomous Region, Inner Mongolia Agricultural University, Hohhot City, China; ^3^Key Laboratory of Mutton Sheep and Goat Genetics and Breeding, Ministry of Agriculture and Rural Affairs, Inner Mongolia Agricultural University, Hohhot City, China; ^4^Inner Mongolia Autonomous Region Agriculture and Animal Husbandry Technology Popularization Center, Hohhot City, China; ^5^East Ujumqin Banner Hishig Animal Husbandry Development Co., Ltd., East Ujumqin Banner, China; ^6^Erdos Agricultural and Animal Husbandry Science Research Institute, Ordos City, China

**Keywords:** attention, computer vision, face recognition, head colors, object detection

## Abstract

**Introduction:**

The facial coloration of sheep is not only a critical characteristic for breed and individual identification but also serves as a significant indicator for assessing genetic diversity and guiding selective breeding efforts.

**Methods:**

In this study, 201 Ujumqin sheep were used as research objects and 1713 head image data were collected. We delineated feature points related to the facial coloration of Ujumqin sheep and successfully developed a head color recognition model (YOLOv8-CBAM) utilizing the YOLOv8 architecture in conjunction with the CBAM attention mechanism.

**Results:**

The model demonstrated impressive performance in recognizing four head color categories, achieving an average precision (mAP) of 97.7% and an F1 score of 0.94. In comparison to YOLOv8n, YOLOv8l, YOLOv8m, YOLOv8s, and YOLOv8x, the YOLOv8-CBAM model enhances average accuracy by 0.5%, 1%, 0.7%, 0.7%, and 1.6%, respectively. Furthermore, when compared to YOLOv3, the improvement is 1%, while YOLOv5n and YOLOv10n show increases of 1.4% and 2.4%, respectively.

**Discussion:**

The findings indicate that the smaller model exhibited superior performance in the facial color recognition task for Ujumqin sheep. Overall, the YOLOv8-CBAM model achieved high accuracy in the head color recognition task, providing reliable technical support for automated sheep management systems.

## 1 Introduction

The color of sheep serves not only as a key indicator for breed identification and classification but also plays a significant role in selective breeding. The Ujumqin sheep has a pure white body coat, while its head coat is primarily white with dark brown markings. Research has demonstrated that the formation of color patches on the heads of sheep is influenced by domestication syndrome and has been inherited over an extended period (1). In the breeding process of Ujumqin sheep, individuals exhibiting a five-point black head color are typically prioritized as breeding stock to prevent the gradual loss of this characteristic due to hybridization or genetic variation, thereby avoiding the mixing of breed traits. Firstly, the artificial identification of head color is highly subjective and can be influenced by individual visual perception, experience level, and assessment preferences. This subjectivity further exacerbates the instability and unreliability of the identification results, increasing the likelihood of misjudgment and leading to low accuracy and efficiency in head color classification. Secondly, with the continuous increase in the global population and the growing demand for mutton, as well as the expansion of meat sheep farming (2), traditional manual identification of head color necessitates that staff closely observe and carefully distinguish each sheep. This process is not only time-consuming and labor-intensive but also poses risks of zoonotic diseases and stress within the sheep flock (3).

With the rapid development of Precision Livestock Farming (PLF), traditional farming practices have undergone a qualitative transformation. The integration of sensors, cameras, machine learning, and image processing technologies has significantly advanced the field of agricultural science. Machine vision, as a non-contact measurement method, effectively mitigates the stress effects on livestock. This detection technique can greatly enhance production efficiency and automation, offering innovative solutions for the modernization of the livestock industry (4–6). The application of computer vision technology in animal husbandry encompasses body sizes analysis (7), behavior monitoring (8), appearance feature recognition (9), and health monitoring (10). This approach provides farmers with a more convenient and effective management tool, serving as a reference for practical applications while substantially reducing subjective errors and labor costs associated with classification (11). The YOLO (You Only Look Once) series (38) is a prominent representative of object detection methods, having undergone multiple iterations that have garnered significant attention in the field due to its high processing speed and accuracy. YOLOv3 employs DarkNet-53 as its backbone network, utilizing residual connections and multi-scale predictions to enhance detection capabilities. It effectively addresses the challenge of varying object sizes by predicting bounding boxes at three different scales (12). YOLOv5 introduces a streamlined architecture that emphasizes efficiency, combining a simple convolutional neural network structure with adaptive anchor box calculations. This approach significantly reduces computational resource usage while maintaining high accuracy. Additionally, YOLOv5 features the Focus layer, which enhances feature extraction by reducing spatial dimensions early in the network (13). YOLOv8 builds upon the architectural principles of YOLOv5, introducing a more complex design that incorporates multiple residual units and branches. This complexity enables YOLOv8 to achieve superior performance metrics, particularly regarding mean average precision (mAP) and detection speed (14, 15). Furthermore, the advancements in YOLOv8 include improved training techniques and optimizations that enhance its robustness for various detection tasks (16). Lastly, YOLOv10 optimizes CSP Darknet and enhances FPN processing of multi-scale features through spatial channel decoupling. It develops dual allocation loss combined with no NMS training to improve detection accuracy and adopts a two-stage training method to promote fine feature learning and detection performance (17).

Currently, various versions of YOLO have been extensively utilized for livestock object detection tasks. Song et al. employed an optimized YOLOv3 model to identify individual faces of Sunite sheep, achieving a mean average precision of 97.20% (18). Furthermore, Zhang et al. introduced an enhanced YOLOv5s model, which attained a mean average precision (mAP@0.5) of 97.8% on a dataset comprising Small Tail Han sheep faces (19). Additionally, Guarnido-Lopez et al. conducted a comparison between the YOLOv8m and YOLOv10m models in monitoring three feeding behaviors of cattle, concluding that both models were effective in predicting the ‘biting’ and ‘chewing’ activities in beef cattle, with an accuracy of approximately 98% (20). This finding suggest that larger parameters of models do not necessarily produce superior results for specific tasks. To identify the most appropriate baseline model for recognizing head color patterns in Ujumqin sheep, a comparative analysis of the training outcomes of multiple YOLO models is warranted.

The attention mechanism plays a crucial role in assigning varying weights to the features of the feature vector. During the training process of the YOLO model, weights are allocated to different regions of the image to minimize redundancy and enhance the accuracy of recognition outcomes (21). For instance, Corkery et al. assessed sheep face recognition using independent component analysis and pre-classifiers, achieving an impressive recognition rate ranging from 95.3 to 96% (22). Similarly, Yadav et al. developed a classifier utilizing YOLOv8 to differentiate between the facial features of sheep and goats, attaining an accuracy of 95.8% in classifying these animals (23). Based on these findings, this study hypothesizes that the YOLO model, enhanced with an attention mechanism, can more effectively recognize the head color patterns of Ujumqin sheep. The objective of this research is to establish a target detection model specifically designed for identifying the head color of Ujumqin sheep. This model aims to classify the head color types of Ujumqin sheep automatically, swiftly, and accurately, thereby providing technical support for the rapid screening of this breed.

## 2 Materials and methods

### 2.1 Data acquisition

The study utilized a fixed channel to capture images of sheep heads, with the images obtained from Hishig Animal Husbandry Development Co., Ltd., located in East Ujumqin Banner, Inner Mongolia Autonomous Region. An RGB camera was installed 0.5 meters above the ground at the channel’s exit. This camera featured an 8-megapixel autofocus high-definition module, equipped with a 1/3.2 inch complementary metal oxide semiconductor (CMOS) IMX179 sensor. It provided a maximum resolution of 3,264 × 2,448 pixels, a field of view of up to 65°, and autofocus capabilities (Kuangda Technology (Shenzhen) Co., Ltd., Shenzhen, Guangdong Province, China). This study focused on 201 Ujumqin sheep, aged between 6 months and 5 years, consisting of 114 rams and 87 ewes. Each sheep was represented by an average of 8–9 images, resulting in a total of 1713 images collected, which included 1,370 images for the training set and 343 images for the validation set.

### 2.2 Data label

**Figure 1:**
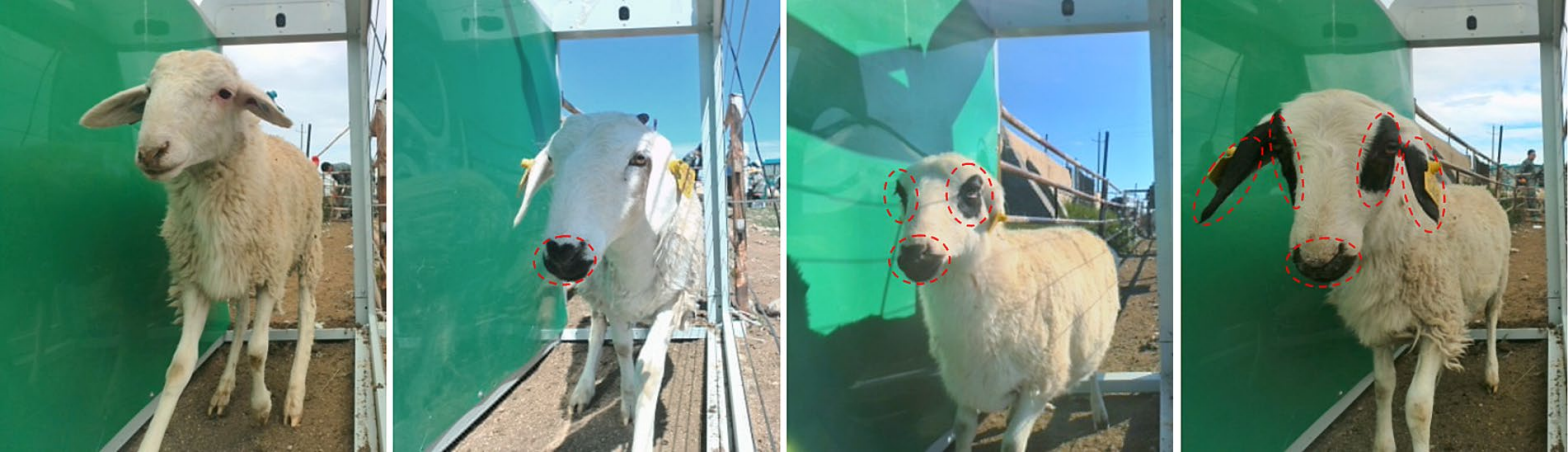
Schematic diagram of Ujumqin sheep head color classification.

Each sheep in the dataset was meticulously identified and recorded. Image annotation was performed using LabelMe. The head data is categorized based on the characteristics of Ujumqin sheep into four groups: pure color, mixed color, three-point black, and five-point black. Figure 1 illustrates the classification standards for the head color of Ujumqin sheep.

1. Pure color: The head is entirely pure white.
2. Mixed color: This category includes heads with a single black spot, a large area of black spots, or irregularly distributed black spots.
3. Three-point black: This classification refers to the presence of black markings around the eyes and near the nose and mouth.
4. Five-point black: This classification is defined by black markings located at the ends of the ears, around the eyes, and near the nose and mouth.

### 2.3 YOLOv8 model optimization

**Figure 2:**
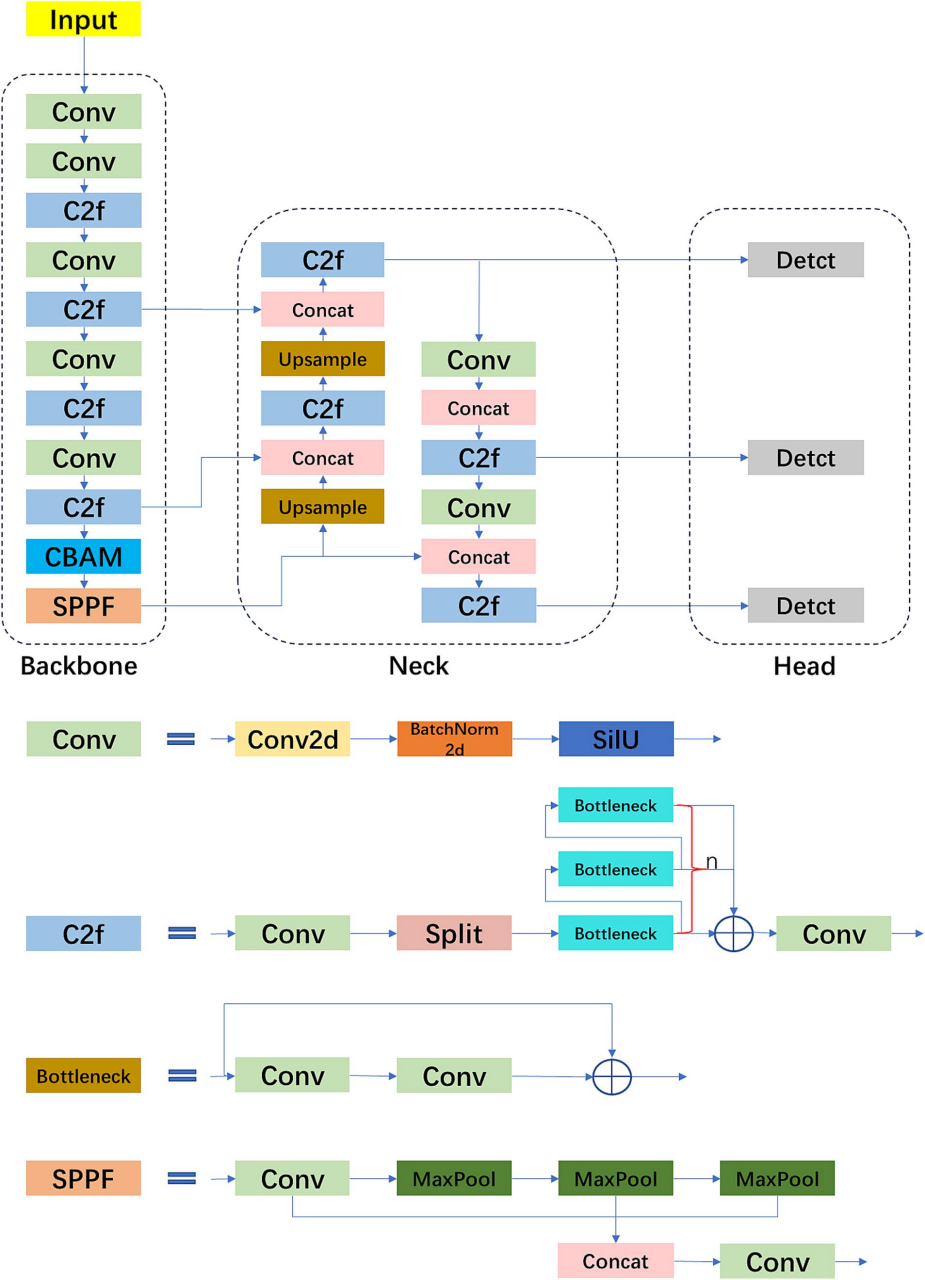
YOLOv8-CBAM model architecture diagram.

Sheep inhabited a complex living environment, and the attention mechanism could enhance the recognition of features associated with the sheep’s head, thereby improving detection accuracy. This study integrated the Convolutional Block Attention Module (CBAM) into the backbone network of YOLOv8, proposing the YOLOv8-CBAM model to distinguish the head colors of Ujumqin sheep. Figure 2 illustrates the architecture of the YOLOv8-CBAM model.

The network structure of YOLOv8-CBAM comprised four primary components: Input, Backbone, Neck, and Head. The Input component was tasked with receiving target images for model training. The Backbone served as the core network of the model, primarily focused on extracting feature information of varying sizes and categories from the images. The Neck was responsible for fusing the features extracted by the Backbone, thereby enhancing the network’s expressive capabilities. The Head utilized the relationships between features to perform tasks such as category prediction and position regression. Key components that constituted the Backbone network included the Conv module, C2f module, Bottleneck module, and Spatial Pyramid Pooling Fusion (SPPF) module.

The Conv module consisted of Conv2d layers and BatchNorm2d layers, and utilized the Sigmoid Linear Unit (SiLU) activation function. The Conv2d layer performed convolution operations, processing the input feature maps with a series of learnable filters to extract local patterns and features. Meanwhile, the BatchNorm2d layer normalized the output of the Conv2d layer, ensuring that feature representations remained stable and consistent throughout the training process. The output of the BatchNorm2d layer was then subject to element-wise application of the SiLU activation function. By introducing this activation function, non-linearity and smoothness were incorporated into feature activations, enhancing the model’s ability to capture complex patterns and ultimately improving overall performance.

The Bottleneck structure played a crucial role in the C2f module by extracting and transforming features of the input data through operations such as feature transformation, branching, and feature fusion, resulting in outputs with greater representational power (17). The C2f module implements feature transformation through two convolution layers, changing the channel count of the input data to 2 * self.c and c2, respectively. It branched the input data into two paths to enhance the network’s non-linearity and representational capability, with one branch outputting directly and the other processed through multiple Bottleneck modules. Finally, feature fusion was achieved by concatenating features from different branches along the channel dimension, enriching the feature representation.

The Bottleneck module improved the network architecture by introducing skip connections, and comprised two convolution layers: the first increased the channel count, while the second decreased it, forming a “bottleneck” structure. The Bottleneck module was used to build deeper network layers to extract high-level features while maintaining low computational complexity, thereby enhancing model performance and accuracy.

**Figure 3:**
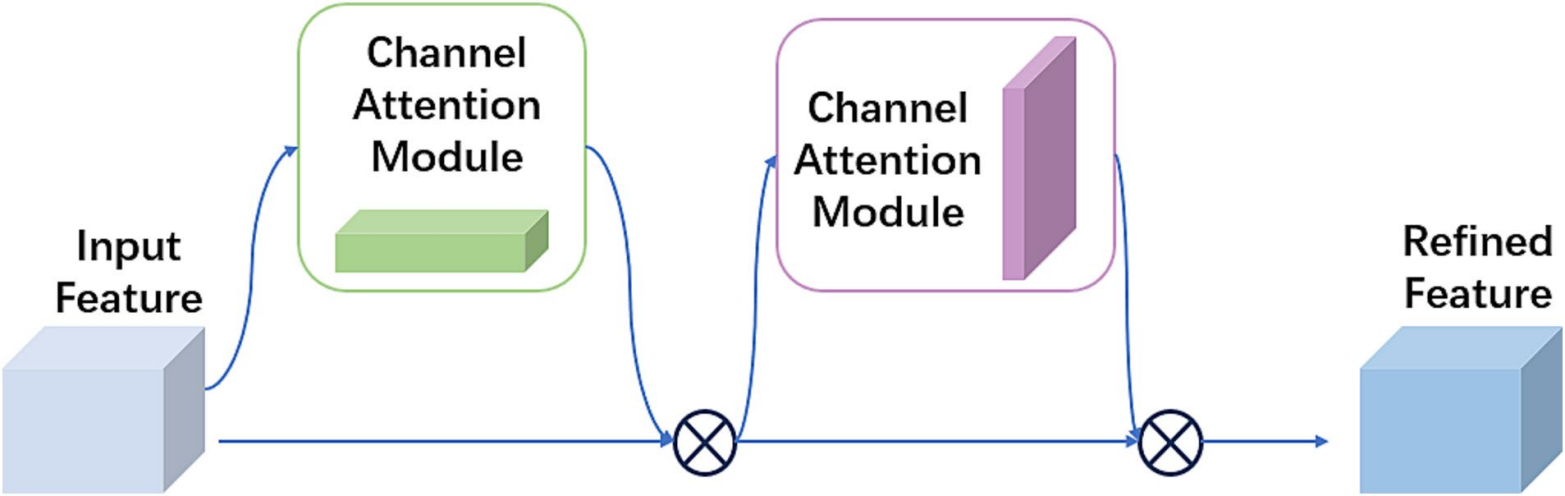
Structure of attention mechanism in CBAM.

The SPPF module was a commonly used pooling component in convolutional neural networks, designed to improve the network’s adaptability to spatial and positional variations in input data, thus enhancing recognition performance. Its fundamental idea was to apply multiple receptive fields of different scales to the same image to capture multi-scale feature information. CBAM consisted of two sub-modules: channel attention and spatial attention modules (24). Figure 3 illustrates the processing flow of this module.

### 2.4 Model parameter

The research utilized an RTX 1060 for training. The supporting Python version was 3.11.9, while PyTorch was at version 2.3.1. The CUDA version employed was 12.1, accompanied by cuDNN version 9.1.1, and the operating system used was Windows 10. A pixel image with a resolution of 640 × 480 served as the input. The training process consisted of 600 epochs, with a batch size of 2. The optimizer used for training was Stochastic Gradient Descent (SGD), with both the initial and final learning rates set at 0.01. The momentum factor was 0.937, and the weight decay coefficient was 0.0005. The training process was terminated when the loss value had not changed significantly for 100 consecutive times.

### 2.5 Evaluation indicator

This study selected the F1 score and the mean average precision (mAP50, mAP50:95) to assess the effectiveness of various models in identifying different behaviors of sheep. Precision denotes the precision of identifying positive samples, while recall indicates the proportion of correctly identified positive samples out of all positive samples. The F1 score represents the harmonic mean of precision and recall, with values ranging from 0 to 1.


$$\mathrm{Precision}=\frac{TP}{TP+FP}$$

$$\mathrm{Reacll}=\frac{TP}{TP+FN}$$

$$F_{1}=2\frac{\mathrm{Pr}\mathrm{ecisio}n^{*}Re\mathrm{call}}{\mathrm{Pr}\mathrm{ecision}+Re\mathrm{call}}$$

where TP is the true positive number, FP is the false positive number, and FN is the false negative number.

Average Precision (AP) is utilized to assess the overall detection performance of a specific category of objects across various confidence thresholds. In this paper, the calculation of AP is accomplished by directly integrating the smoothed curve. Mean Average Precision (mAP) represents the average of the individual AP values computed for different categories. The term mAP50 denotes the model’s average precision when accounting for the overlap area ratio, known as Intersection over Union (IoU), between the predicted and ground truth boxes at a threshold of 0.5. mAP50:95 refers to the results derived with a step size of 0.05, encompassing IoU thresholds that range from 0.5 to 0.95. This metric offers a thorough evaluation of the model’s capabilities in object detection tasks. Collectively, these evaluation metrics gauge the effectiveness and accuracy of the model in object detection and quantification, calculated using the following formula:


$$AP=\overset{1}{\underset{0}{\int }}P\left(R\right)dR$$

$$\mathrm{mAP}=\frac{\sum_{1}^{n}\left(AP\right)}{n}$$

Where R stands for recall rate. *n* represents the number of categories.

## 3 Results

### 3.1 YOLOv8n-cbam model training results

**Figure 4:**
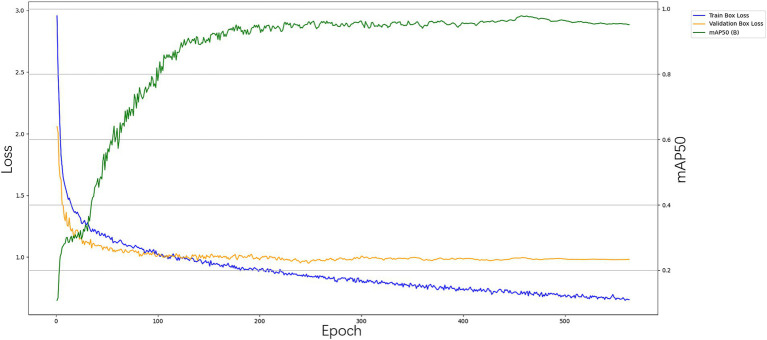
YOLOv8n-cbam model training diagram.

During the first 100 training rounds, the model’s loss value decreased rapidly, indicating a significant improvement in performance during the initial learning phase. Concurrently, both accuracy and recall rates exhibited a marked increase. From the 100th to the 200th round, the loss value began to decline at a slower rate, suggesting that the model was gradually approaching a local optimal solution. After 200 training rounds, the loss value stabilized, and both accuracy and recall rates reached a plateau, indicating that the model’s performance had converged. The model training diagram is presented in Figure 4.

**Figure 5:**
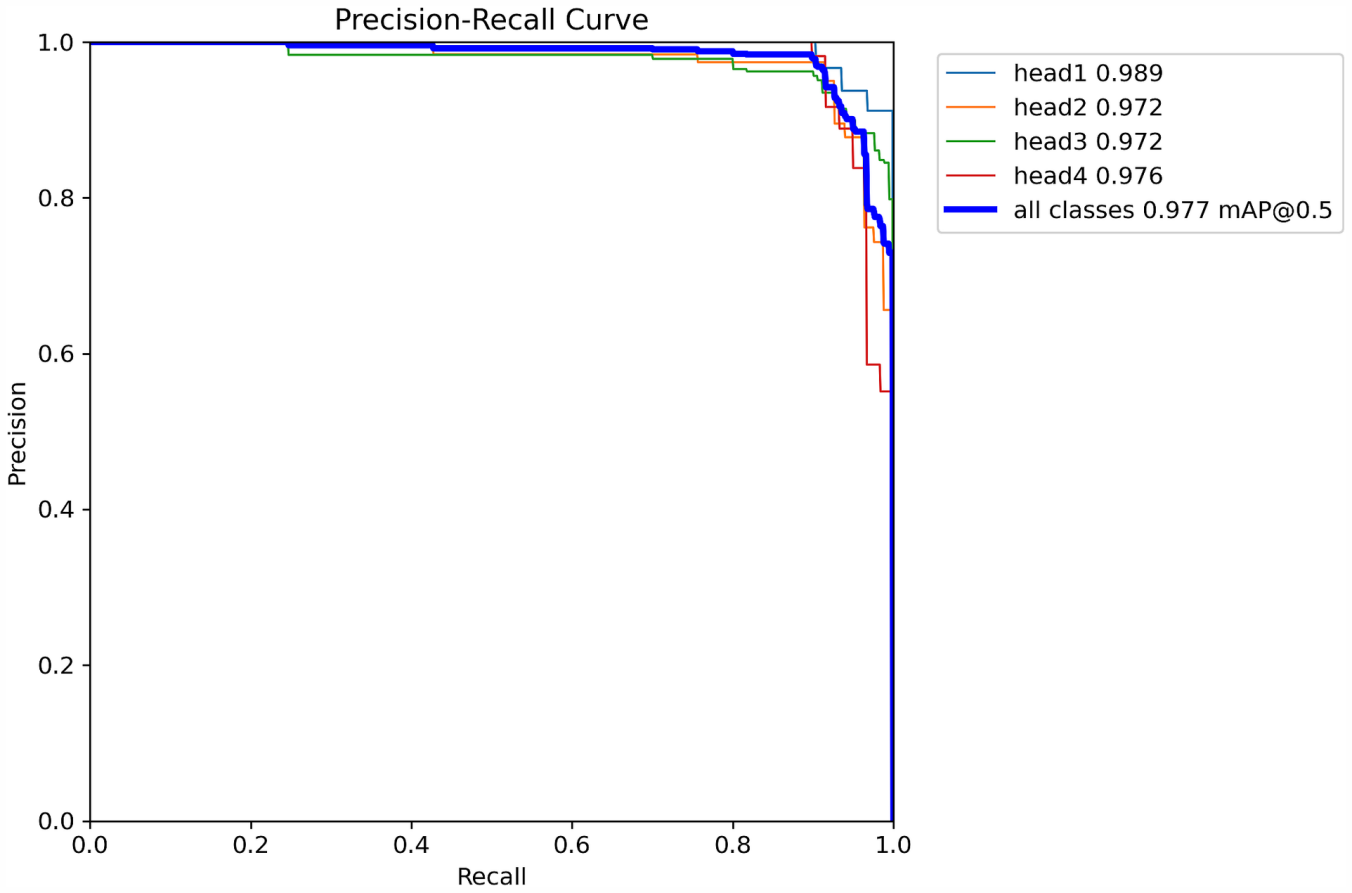
PR curve of YOLOv8n-CBAM model.

Figure 5 illustrates the precision-recall (PR) curve of the YOLOV8n-CBAM model, where head1, head2, head3, and head4 correspond to four distinct head colors: pure color, five-point black, mixed color, and three-point black, respectively. The accuracy rates for these colors are as follows: pure color at 98.9%, five-point black at 97.2%, mixed color at 97.2%, and three-point black at 97.6%. The average accuracy across the four categories is 97.7%.

### 3.2 YOLO model comparison experiment results

In the task of identifying facial patterns among different families of sheep, the accuracy of each version of the YOLOv8 model exhibits notable differences. The comparison results between the improved model proposed in this study and the original YOLOv8 model are presented in Table 1. The mAP50% accuracy for YOLOv8n, YOLOv8l, YOLOv8m, YOLOv8s, and YOLOv8x are 0.972, 0.967, 0.970, 0.970, and 0.961, respectively. The mAP50:95% accuracy values are 0.744, 0.736, 0.732, 0.731, and 0.725, respectively. YOLOv8n, with an accuracy rate of 0.972. Although YOLOv8x, with an accuracy of 0.961, possesses stronger feature extraction capabilities, it does not achieve the same level of accuracy as the smaller model when applied to the sheep head color dataset. Notably, YOLOv8s achieves the highest F1 score of 0.94. The mAP50% of the improved model presented in this article is 0.977, while the mAP50:95% accuracy is 0.745. The improved model, YOLOv8-CBAM, exhibits higher accuracy and F1 score compared to the various versions of the YOLOv8 model in classifying sheep head colors.

**Table 1:** Improved model performance analysis.

Model	AP50%				mAP50%	mAP50:95%	F1-score
	Head1	Head2	Head3	Head4			
YOLOv8n	0.971	0.967	0.981	0.969	0.972	0.744	0.93
YOLOv8l	0.972	0.963	0.974	0.958	0.967	0.736	0.93
YOLOv8m	0.962	0.970	0.973	0.975	0.970	0.732	0.93
YOLOv8s	0.964	0.976	0.978	0.961	0.970	0.731	0.94
YOLOv8x	0.975	0.971	0.968	0.932	0.961	0.725	0.91
YOLOv8n-CBAM	0.989	0.972	0.972	0.976	0.977	0.745	0.94

Head1 = pure color; Head2 = Five-point black; Head3 = mixed color; Head4 = three-point black; AP50% = the average accuracy obtained in the case where the IoU threshold is set to 50%; mAP50 = computes the area under the precision-recall curve across multiple classes, calculated at an intersection over union threshold of 50%; mAP50-95: the average of the mean average precision calculated at varying IoU thresholds, ranging from 50 to 95%; F1-score = the harmonic average of Precision and Recall.

The study compared the performance of the proposed algorithm against the YOLOv3, YOLOv5n, YOLOv8n, and YOLOv10n models using the Ujumqin sheep head color dataset. All models were trained and tested within the same experimental environment. The accuracy comparison results for each model on the dataset, measured at mAP50% and mAP50:95%, are presented in Table 2. YOLOv8n outperformed YOLOv3, YOLOv5n, and YOLOv10n in both accuracy and F1 score. In comparison to the other models, the improved model proposed in this article demonstrated enhancements in mAP50%, mAP50:95%, and F1 score.

**Table 2:** Different models compare experimental results.

Model	AP50%				mAP50%	mAP50:95%	F1-score
	Head1	Head2	Head3	Head4			
YOLOv3	0.987	0.974	0.966	0.94	0.967	0.713	0.92
YOLOv5n	0.972	0.958	0.968	0.955	0.963	0.732	0.93
YOLOv8n	0.971	0.967	0.981	0.969	0.972	0.744	0.93
YOLOv10n	0.965	0.956	0.962	0.93	0.953	0.724	0.92
YOLOv8n-CBAM	0.989	0.972	0.972	0.976	0.977	0.745	0.94

Head1 = pure color; Head2 = Five-point black; Head3 = mixed color; Head4 = three-point black; AP50% = the average accuracy obtained in the case where the IoU threshold is set to 50%; mAP50 = computes the area under the precision-recall curve across multiple classes, calculated at an intersection over union threshold of 50%; mAP50-95: the average of the mean average precision calculated at varying IoU thresholds, ranging from 50 to 95%; F1-score = the harmonic average of Precision and Recall.

## 4 Discussion

With the advancement of intensive breeding systems, the demand for contactless individual facial recognition technology is increasing. However, there is currently a paucity of research on feature point recognition specific to certain sheep species, with most studies primarily concentrating on behavioral detection (25, 26). This study introduces the Ujumqin sheep facial recognition model, YOLOv8-CBAM. By incorporating the CBAM module prior to the SPP module in the feature extraction layer (Backbone) of YOLOv8, the model enhances its focus on the target area while minimizing the interference from irrelevant information. The average recognition accuracy of the enhanced YOLOv8n-CBAM model in this study was 97.7%, with an F1 score of 0.94. Compared to the YOLOv8 series models, the YOLOv8-CBAM model demonstrated a significant increase in mAP50% accuracy, with YOLOv8n improving by 0.5%, YOLOv8l by 1%, YOLOv8m by 0.7%, YOLOv8s by 0.7%, and YOLOv8x by 1.6%. When compared to other YOLO models, improvements were also notable: YOLOv3 increased by 1%, YOLOv5n by 1.4%, and YOLOv10n by 2.4%. The addition of CBAM enabled the model to better recognize the facial features of sheep, thereby enhancing prediction accuracy. Related research has indicated that incorporating an attention mechanism can further improve model accuracy. For instance, Jiang et al. proposed a CBAM-YOLOv7 algorithm that enhances the attention mechanism by integrating three CBAM modules into the YOLOv7 backbone network, thereby improving the network’s feature extraction capability (27). Lei et al. enhanced YOLOv5 by incorporating the CBAM and deconvolution, resulting in the YOLOv5-CBAM + TC model. This model demonstrates superior accuracy, recall, and mAP compared to both the original YOLOv5 and the YOLOv5-SD model, which integrates a small target detection layer (28). Similarly, Hao et al. introduced the CBAM module and the Spatial Pyramid Pooling (SPP) module to YOLOv3, creating the YOLOv3-SC network specifically for pig target detection. This network achieved a remarkable mAP of 99.24% with a detection time of just 16 ms. When compared to YOLOv1, YOLOv2, Faster R-CNN, and YOLOv3, the mAP for pig recognition increased by 2.31, 1.44, 1.28, and 0.61%, respectively (29). Furthermore, Xue et al. combined Convolutional Neural Networks (CNNs) and Transformer architectures, integrating the CBAM module to develop the CAT-CBAM-Net model. This model was evaluated against EfficientNet-B0, VGG-16, ResNet-18, AlexNet, ViT-base-32, and Swin-Transformer, showing significant improvements (30).

This study found that the recognition accuracy of YOLOv8n exceeds that of the YOLOv8 model, which has a greater number of network layers. This suggests that, in the context of detecting sheep facial features, deeper convolutional layers may result in increased misclassifications during image classification, while also requiring more processing time. Additionally, the demand for more computing resources contributes to a longer model inference time (31). YOLOv8-CBAM, a modified version of the smaller model, demonstrates superior accuracy, F1 score, and mean Average Precision (mAP) in the classification of sheep head color when compared to various versions of the YOLOv8 model. Ali et al. conducted a comparative evaluation of model performance across YOLOv5l, YOLOv5m, YOLOv5n, YOLOv5s, and YOLOv5x, concluding that YOLOv5n achieved the highest mean Average Precision (mAP) for both small and large instances, with a score of 77.40% (32). Gamani et al. assessed the performance of various configurations of the YOLOv8 model, finding that YOLOv8n attained the fastest inference speed at 24.2 milliseconds, outperforming YOLOv8s, YOLOv8m, YOLOv8l, and YOLOv8x, which recorded speeds of 33.0 milliseconds, 44.3 milliseconds, 53.6 milliseconds, and 62.5 milliseconds, respectively (33). Casas et al. compared YOLOv8n, YOLOv8s, YOLOv8m, YOLOv8l, and YOLOv8x in their research. Their results indicated that YOLOv8n achieved the highest verification accuracy and training accuracy, with values of 0.644 and 0.703, respectively (34).

In the comparison of various YOLO versions, the study found that YOLOv8n outperforms YOLOv3 (35), YOLOv5n, and YOLOv10n (17). These results may be attributed to the structural differences among the models of different YOLO versions. YOLOv8 is particularly suitable for head classification research on sheep; therefore, it is essential to select a model that aligns with the characteristics of the dataset. The YOLOv3 model is primarily composed of Darknet-53 and Feature Pyramid Network (FPN), and it does not include a pooling layer. The backbone network consistently employs 53 convolutional layers, hence the name Darknet-53. The FPN facilitates the model’s ability to perform multi-scale predictions. As an earlier version of the YOLO model, YOLOv3 lacks several processing modules, resulting in some target detection outcomes that are inferior to those of YOLOv8. Yang et al. concluded in their experiment on pest detection in wild cotton fields that YOLOv8 demonstrated superior performance in terms of Precision, Recall, and mAP compared to YOLOv3 (24).

The YOLOv5, YOLOv8, and YOLOv10 models share a similar structural framework, yet they exhibit notable differences. In contrast to YOLOv5, YOLOv8 primarily replaces the C3 module in both the Backbone and Neck with the C2f module. This substitution results in lower computational requirements compared to the C3 module, significantly enhancing both convergence speed and overall performance. Additionally, YOLOv8 replaces the SPP module in the Backbone with the more efficient SPPF module, and it substitutes the coupled head in the detection head with a decoupled head. These changes not only improve model accuracy but also expedite network convergence. Supporting this, Ma et al. observed that the Precision, Recall, mAP50, and mAP50-95 values for YOLOv5n were lower than those for YOLOv8n in the context of pepper target detection (36).

Compared to YOLOv8, the most significant changes in YOLOv10 include the introduction of the PSA layer and the CIB layer, alongside the removal of Non-Maximum Suppression (NMS). The PSA module, which is positioned after the SPPF, integrates a 1 × 1 convolution, a multi-head self-attention module, and a feed-forward network. Additionally, the bottleneck structure within part of the C2F module has been modified to adopt the CIB structure, resulting in the creation of the C2FCIB module for large-core convolution. While YOLOv8 employs an anchor-free approach and utilizes NMS for post-processing following predictions, YOLOv10 implements dual label allocation. In contrast to one-to-many assignment, the one-to-one matching method assigns a single prediction to each true value, thereby eliminating the need for NMS in post-processing. However, this approach may lead to insufficient supervision, which can negatively impact accuracy and convergence speed. Consequently, the incorporation of the new modules may cause YOLOv10 to perform worse than YOLOv8 on certain tasks. Gong et al. compared the evaluation indicators of Recall and mAP50-95 for YOLOv8n in the context of target detection for sika deer posture recognition. Their findings indicated that YOLOv10 performed worse than YOLOv8s in terms of Precision, Recall, mAP50, and mAP50-95 (37). Similarly, Ma et al. found that YOLOv10 underperformed compared to YOLOv8n across the same metrics in pepper target detection. In summary, YOLOv8n demonstrates superior performance compared to YOLOv3, YOLOv5n, and YOLOv10n, likely due to the specific relationship between the tasks and the model structure (36). While this study successfully achieved high-precision discrimination among head families of Ujumqin sheep, it also offers a novel solution for the efficient screening of these sheep. After introducing the attention mechanism, the YOLO model can improve the recognition ability of the head color mode. However, to facilitate portable identification of grazing groups and to address limitations related to varying lighting conditions and shooting distances, further research is needed to enhance the model’s robustness and stability.

## 5 Conclusion

This study compares different versions of the YOLO model and proposes an improved algorithm based on YOLOv8n, named YOLOv8n-CBAM. The algorithm guides the neural network to focus on key feature regions while suppressing irrelevant information, significantly enhancing detection accuracy. The study has successfully achieved automated, rapid, and precise classification of the head color types of Ujumqin sheep, providing technical support for the efficient selection of this breed. To improve the model’s adaptability in practical applications and expand its potential for head color recognition in other sheep breeds, future research should focus on enhancing the model’s robustness under varying lighting conditions and shooting distances, further optimizing its stability and accuracy, while also improving real-time performance to facilitate its application in complex environments.

## Data Availability

Data supporting the results of this study are available from the corresponding author upon reasonable request. Requests to access the datasets should be directed to liuzh7799@163.com.
